# Prognostic Role of Neutrophil to High-Density Lipoprotein Cholesterol Ratio for All-Cause and Cardiovascular Mortality in the General Population

**DOI:** 10.3389/fcvm.2022.807339

**Published:** 2022-02-08

**Authors:** Ming Jiang, Jinyu Sun, Huayiyang Zou, Menghuan Li, Zhenyang Su, Wei Sun, Xiangqing Kong

**Affiliations:** ^1^Department of Cardiology, The First Affiliated Hospital of Nanjing Medical University, Nanjing, China; ^2^Department of Cardiology, The Medical School of Southeast University, Nanjing, China; ^3^Gusu School, Nanjing Medical University, Suzhou, China

**Keywords:** neutrophil, high-density lipoprotein cholesterol, all-cause mortality, cardiovascular mortality, NHANES

## Abstract

**Background:**

Neutrophil counts to high-density lipoprotein cholesterol ratio (NHR), a composite marker of inflammation and lipid metabolism, has been considered as a predictor of clinical outcomes in patients with acute ischemic stroke and acute myocardial infarction. However, the predictive value of NHR for all-cause and cardiovascular mortality in the general population remains unclear.

**Methods:**

Our study population comprised 34,335 adults in the United States obtained from the National Health and Nutrition Examination Survey (NHANES) (1999–2014) and were grouped in accordance with tertiles of NHR. Kaplan–Meier curves and log-rank test were used to investigate the differences of survival among groups. Multivariate Cox regression, restricted cubic spline analysis, and subgroup analysis were applied to explore the relationship of NHR with all-cause and cardiovascular mortality.

**Results:**

The mean age of the study cohort was 49.6 ± 18.2 years and 48.4% were men. During a median follow-up of 82 months, 4,310 (12.6%) all-cause deaths and 754 (2.2%) cardiovascular deaths occurred. In a fully-adjusted Cox regression model, participants in the highest tertile had 29% higher hazard of all-cause mortality than those in the lowest tertile [hazard ratio (HR) = 1.29, 95% *CI*: 1.19–1.41]. For cardiovascular mortality, the continuously increased HR with 95% *CI*s among participants in the middle and highest tertile were 1.30 (1.06–1.59) and 1.44 (1.17–1.78), respectively. The restricted cubic spline curve indicated that NHR had a non-linear association with all-cause mortality (*p* for non-linearity < 0.001) and a linear association with cardiovascular mortality (*p* for non-linearity = 0.553).

**Conclusion:**

Increased NHR was a strong and independent predictor of all-cause and cardiovascular mortality in the general population.

## Introduction

Neutrophil to high-density lipoprotein cholesterol ratio (NHR) is a composite marker of inflammation and lipid metabolism, which allows simultaneous studies on the separate impact and more insights into the interaction (1). The two hematological parameters are inexpensive, easy to measure, and well-standardized. These features make NHR a promising clinical indicator. NHR is reportedly associated with the prevalence of metabolic syndrome (2). However, the effect of NHR on all-cause and cardiovascular mortality has received limited attention.

It is known that chronic inflammation and abnormal lipid metabolism play crucial roles in the pathophysiology of atherosclerosis, leading to adverse cardiovascular events and mortality (3). As proinflammatory cells, neutrophils have been recently recognized to participate in the various stages of atherosclerosis, which can aggravate endothelial dysfunction, recruit monocytes into atherosclerotic lesions, activate macrophages, promote foam cell formation, and contribute to plaque destabilization (4). By contrast, high-density lipoprotein cholesterol (HDL-C) has been considered as a protective factor against atherosclerosis. The major cardioprotective mechanisms include reverse cholesterol transport, antioxidative properties, and anti-inflammatory effect in the endothelium (5). Previous epidemiologic studies have demonstrated that HDL-C is an important predictor for mortality and cardiovascular mortality (6, 7).

Only few retrospective studies have investigated the correlation of NHR and short-term prognosis after intravenous thrombolysis in patients with acute ischemic stroke (8) and long-term mortality in patients with acute myocardial infarction (1, 9). To our knowledge, there is no study yet on the association between NHR and long-term mortality in the general population. Therefore, we aimed to assess the predictive value of NHR for all-cause and cardiovascular mortality among the general adult population of the United States using the clinical data available from the National Health and Nutrition Examination Survey (NHANES).

## Methods

### Study Population

**Figure 1:**
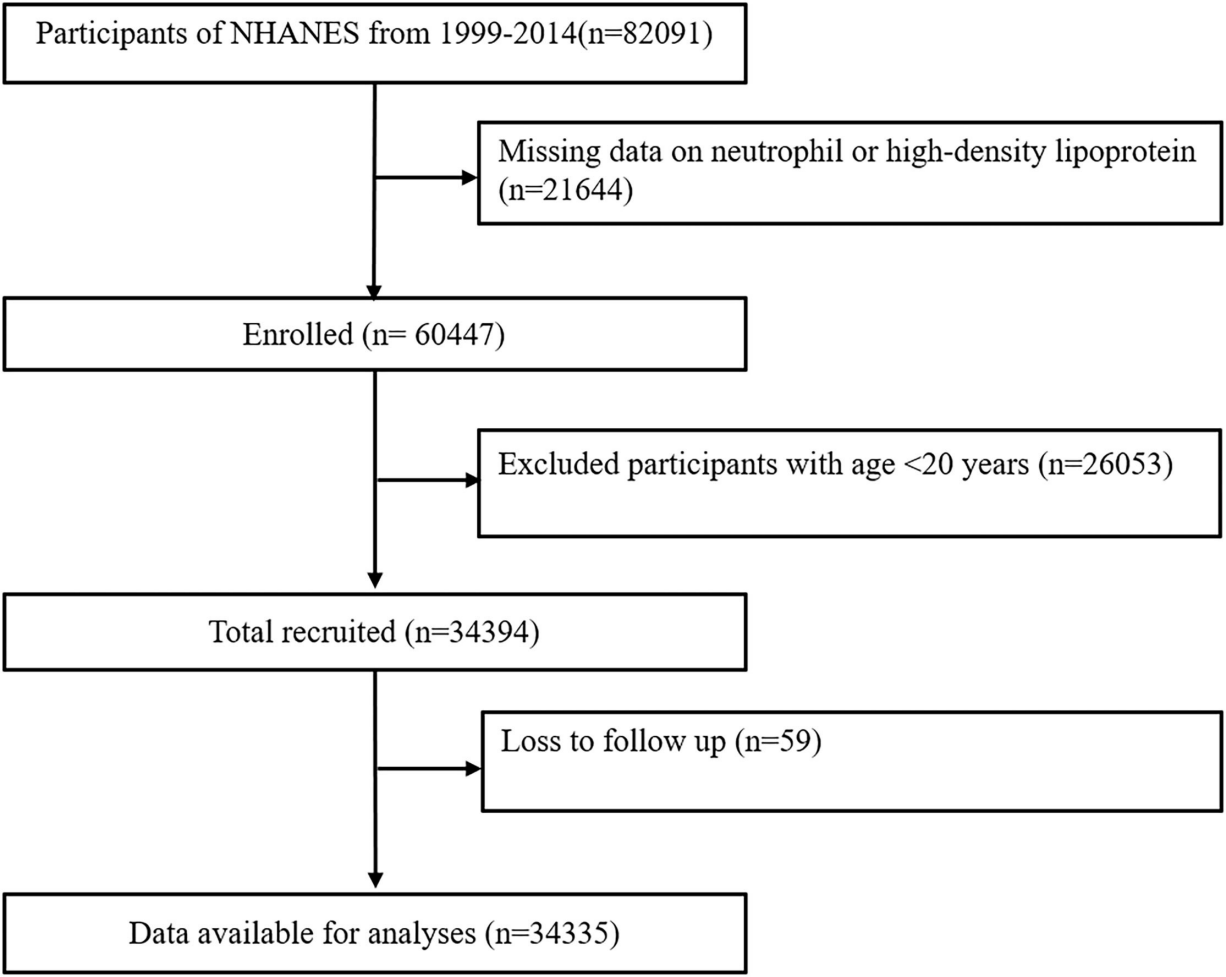
Study flowchart. NHANES, National Health and Nutrition Examination Survey.

The NHANES is a continuous program conducted by the National Center for Health Statistics (NCHS). The sample of the NHANES survey is selected to represent the non-institutionalized civilian population of the United States using a complex, multistage, probability sampling design. The 1999–2014 survey cycles included a total of 82,091 participants. Our study included participants in whom neutrophil count and high-density lipoprotein cholesterol data were available (*n* = 60,447). After excluding participants aged <18 years (*n* = 26,053) and those lost to follow-up (*n* = 59), a total of 34,335 participants were included for further analysis (Figure 1). The survey protocol was approved by the NCHS research ethics review board, and all participants provided written informed consent.

### Assessment of Exposure

Neutrophil to high-density lipoprotein cholesterol ratio was calculated as the neutrophil count (10^3^ cells/μl) divided by the HDL-C (mg/dl) value, both of which were acquired from laboratory tests. Blood specimens were collected following established venipuncture protocol and procedures. The UniCel DxH 800 Analyzer was used to measure the neutrophil absolute number. HDL-C was measured on the Roche modular P and Roche Cobas 6000 chemistry analyzers. This study modeled the tertiles of the exposure distribution to assess the potential association between NHR and the target outcomes.

### Assessment of Covariates

Demographic information, such as age, sex, race/ethnicity, and education level were collected by questionnaires. Smokers were defined as participants who reported smoking more than 100 cigarettes during their lifetime, and those who consumed at least 12 drinks in the past 12 months were classified as alcohol users (10). Self-reported personal interview data provided the medical history of diabetes mellitus, hypertension, heart failure, coronary heart disease, stroke, and cancer. Body mass index (BMI) was calculated as the weight in kilograms divided by the square of height in meters, which was obtained from the body measurements. Systolic blood pressure (SBP) and diastolic blood pressure (DBP) were determined as the average values of three consecutive blood pressure readings after resting quietly for 5 min. Albumin and serum creatinine test results were obtained from laboratory tests. The estimated glomerular filtration rate (eGFR) was calculated according to the MDRD formula (11).

### Ascertainment of Outcomes

The outcomes of this study were all-cause mortality and cardiovascular mortality. Mortality status and underlying cause of death were identified by the NHANES-linked National Death Index record. The definition of cardiovascular mortality was based on the International Classification of Diseases, 10th Revision (ICD-10) (12). The public-use linked mortality file for the 1999–2014 NHANES provided follow-up time from the date of survey participation through December 31, 2015. More detailed information about the linkage methodology and analytic guidelines are available on the NCHS data linkage webpage (13).

### Statistical Analysis

Baseline characteristics were described in accordance with the tertiles of NHR (<0.065, 0.065–0.100, and >0.100). Continuous variables were expressed as the mean ± SD, and categorical variables were expressed as numbers with percentages. Comparisons between the tertiles of NHR were performed using one-way ANOVA for continuous variables and the chi-square test for categorical variables. The differences of survival rates according to NHR tertiles were analyzed by Kaplan–Meier survival curves and log-rank test. Multivariate Cox regression models were built to investigate the independent association of NHR with all-cause and cardiovascular mortality. Model 1 was a crude model with no confounders. Model 2 was adjusted for age, sex, race, education status, smoker, and alcohol user. Model 3 was adjusted for all variables in model 2 and other confounders, such as diabetes mellitus, hypertension, heart failure, coronary heart disease, stroke, cancer, BMI, albumin, eGFR, antihypertensive drugs, and antidiabetic drugs. Restricted cubic spline regression models were used to explore any non-linear relationship of NHR with all-cause and cardiovascular mortality. For subgroup analysis, we tested the results stratified by age, sex, and history of diabetes or hypertension from fully-adjusted regression models. Statistical analyses were performed using Stata version 14.0, R version 3.5.3 and the EmpowerStats software (http://www.empowerstats.com). The value of *p* < 0.05 was considered to indicate statistical significance.

## Results

### Baseline Characteristics

Table 1 presents the baseline characteristics of 34,335 participants finally included in this study. The average age of this study population was 49.6 ± 18.2 years and 48.4% were men. All baseline confounders had significant differences among NHR tertiles (all *p* < 0.01), except for SBP, DBP, and cancer. During a median follow-up of 82 months, 4,310 (12.6%) all-cause deaths and 754 (2.2%) cardiovascular deaths occurred.

**Table 1:** Baseline characteristics of the study population.

	**Total**	**Neutrophil to high-density lipoprotein ratio (NHR)**			***P*-value**
		**Tertile1**	**Tertile2**	**Tertile3**	
		** <0.065**	**0.065-0.100**	**>0.100**	
Number		11,439	11,266	11,630	
Age, year	49.6 ± 18.2	50.8 ± 18.0	49.8 ± 18.3	48.2 ± 18.3	<0.001
Male, *n* (%)	16,601 (48.4%)	4,568 (39.9%)	5,510 (48.9%)	6,523 (56.1%)	<0.001
Race, *n* (%)					<0.001
Mexican American	6,187 (18.0%)	1,471 (12.9%)	2,286 (20.3%)	2,430 (20.9%)	
Non-Hispanic White	16,133 (47.0%)	4,781 (41.8%)	5,261 (46.7%)	6,091 (52.4%)	
Non-Hispanic Black	6,691 (19.5%)	3,454 (30.2%)	1,881 (16.7%)	1,356 (11.7%)	
Other races	5,324 (15.5%)	1,733 (15.1%)	1,838 (16.3%)	1,753 (15.1%)	
Education status, *n* (%)					<0.001
<9th grade	4,352 (12.7%)	1,213 (10.6%)	1,519 (13.5%)	1,620 (13.9%)	
9–11th grade	5,411 (15.8%)	1,651 (14.4%)	1,676 (14.9%)	2,084 (17.9%)	
High school	7,957 (23.2%)	2,420 (21.2%)	2,602 (23.1%)	2,935 (25.2%)	
College	9,445 (27.5%)	3,169 (27.7%)	3,065 (27.2%)	3,211 (27.6%)	
Graduate	7,170 (20.9%)	2,986 (26.1%)	2,404 (21.3%)	1,780 (15.3%)	
Smoker, *n* (%)	15,975 (46.5%)	4,548 (39.8%)	5,018 (44.5%)	6,409 (55.1%)	<0.001
Alcohol user, *n* (%)	24,198 (70.5%)	7,999 (69.9%)	7,879 (69.9%)	8,320 (71.5%)	0.008
Diabetes mellitus, *n* (%)	24,198 (70.5%)	855 (7.5%)	1,257 (11.2%)	1,766 (15.2%)	<0.001
Hypertension, *n* (%)	11,722 (34.1%)	3,599 (31.5%)	3,807 (33.8%)	4,316 (37.1%)	<0.001
Heart failure, *n* (%)	1,093 (3.2%)	243 (2.1%)	326 (2.9%)	524 (4.5%)	<0.001
Coronary heart disease, *n* (%)	1,450 (4.2%)	314 (2.7%)	469 (4.2%)	667 (5.7%)	<0.001
Stroke, *n* (%)	1,240 (3.6%)	326 (2.8%)	390 (3.5%)	524 (4.5%)	<0.001
Cancer, *n* (%)	3,078 (9.0%)	1,070 (9.4%)	998 (8.9%)	1,010 (8.7%)	0.183
Body mass index, kg/m^2^	28.8 ± 6.6	26.9 ± 5.8	28.7 ± 6.2	30.7 ± 7.1	<0.001
Systolic BP, mmHg	124.6 ± 19.6	124.5 ± 20.4	124.6 ± 19.5	124.6 ± 18.9	0.765
Diastolic BP, mmHg	70.0 ± 13.4	70.1 ± 12.9	70.0 ± 13.2	70.0 ± 14.1	0.571
Neutrophil count,10^3^/μl	4.32 ± 1.81	2.92 ± 0.84	4.15 ± 0.98	5.85 ± 1.95	<0.001
HDL-C, mg/dl	52.54 ± 15.85	64.08 ± 16.17	51.62 ± 11.75	42.08 ± 10.45	<0.001
Albumin, g/dL	4.3 ± 0.4	4.3 ± 0.3	4.3 ± 0.4	4.2 ± 0.4	<0.001
eGFR, ml/min/1.73 m^2^	95.1 ± 32.8	92.6 ± 30.4	95.6 ± 32.1	97.1 ± 35.3	<0.001
Antihypertensive drugs, n (%)	8,773 (26.9%)	2,644 (24.3%)	2,887 (27.0%)	3,242 (29.5%)	<0.001
Antidiabetic drugs, n (%)	3,029 (9.0%)	638 (5.6%)	984 (8.9%)	1,407 (12.3%)	<0.001
Outcomes, n (%)					
All-cause mortality	4,310 (12.6%)	1,262 (11.0%)	1,372 (12.2%)	1,676 (14.4%)	<0.001
Cardiovascular mortality	754 (2.2%)	187 (1.6%)	260 (2.3%)	307 (2.6%)	<0.001

*BP, blood pressure; HDL-C, high-density lipoprotein cholesterol ratio; eGFR, estimated glomerular filtration rate*.

*Values are mean ± SDs or number (%)*.

### Association of NHR With All-Cause Mortality

**Figure 2:**
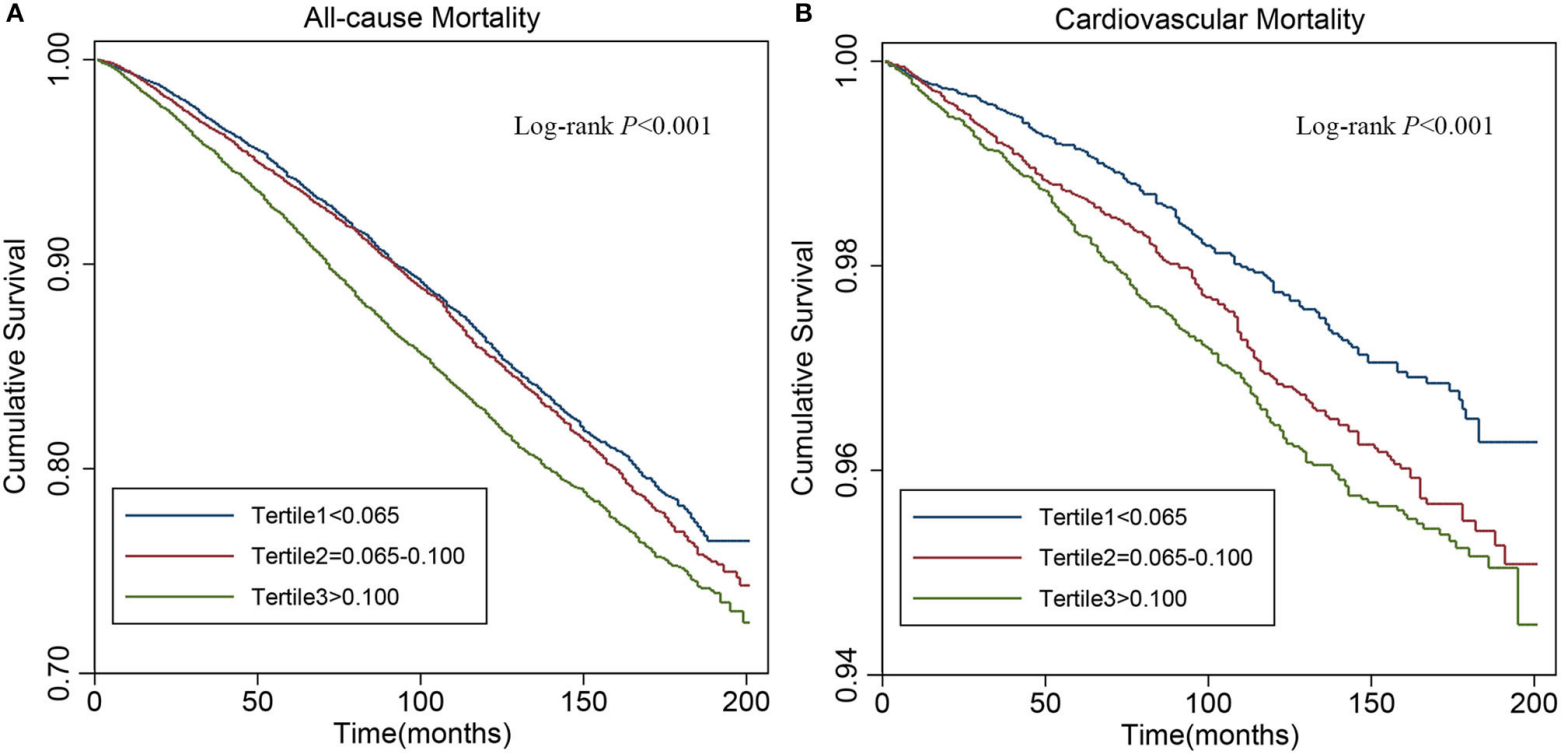
Kaplan–Meier analysis for all-cause **(A)** and cardiovascular **(B)** mortality by tertiles of neutrophil to high-density lipoprotein cholesterol ratio (NHR).

**Figure 3:**
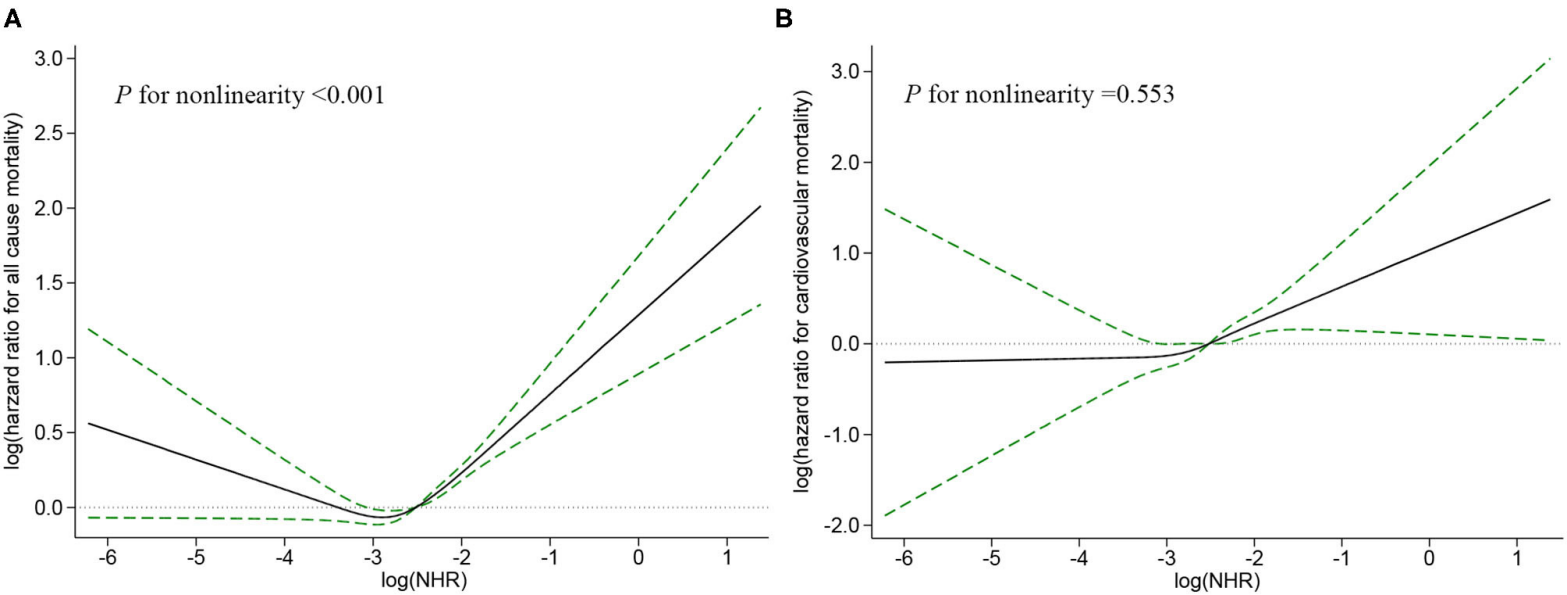
Restricted cubic spline plots of associations between NHR with all-cause **(A)** and cardiovascular **(B)** mortality in the general population. Analysis was adjusted for age, sex, race, education status, smoker, alcohol user, diabetes mellitus, hypertension, heart failure, coronary heart disease, stroke, cancer, body mass index (BMI), albumin, estimated glomerular filtration rate (eGFR), antihypertensive drugs, and antidiabetic drugs. The solid line and dashed line represent the log-transformed hazard ratios and corresponding 95% *CI*s, respectively.

For all-cause mortality, Kaplan–Meier survival curves showed statistically significant differences in survival probabilities among the NHR tertiles (log-rank test, *p* < 0.001, Figure 2A). In a fully-adjusted Cox regression model (Table 2), participants in the highest tertile had 29% higher risk of death from any cause than those in the lowest tertile [hazard ratio (*HR*) = 1.29, 95% *CI*: 1.19–1.41], while the middle tertile did not differ significantly from the lowest tertile (*HR* = 1.07, 95% *CI*: 0.98–1.16). The association between NHR and all-cause mortality was non-linear and U-shaped according to restricted cubic spline models (Figure 3A), and the test for non-linearity was significant (*p* < 0.001).

**Table 2:** The multivariate cox regression analysis of NHR with cause-specific mortality.

**Outcomes**	**Model 1**	***P***	**Model 2**	***P***	**Model 3**	***P***
	**HR (95%CI)**		**HR (95%CI)**		**HR (95%CI)**	
All-cause mortality						
NHR tertile						
Tertile1	Reference		Reference		Reference	
Tertile2	1.06 (0.98, 1.14)	0.140	1.07 (0.99, 1.16)	0.085	1.07 (0.98, 1.16)	0.124
Tertile3	1.27 (1.18, 1.37)	<0.001	1.42 (1.31, 1.53)	<0.001	1.29 (1.19, 1.41)	<0.001
*P* for trend	<0.001		<0.001		<0.001	
Cardiovascular mortality					
NHR tertile						
Tertile1	Reference		Reference		Reference	
Tertile2	1.36 (1.13, 1.64)	0.001	1.39 (1.15, 1.69)	0.001	1.30 (1.06, 1.59)	0.011
Tertile3	1.58 (1.32, 1.89)	<0.001	1.80 (1.48, 2.18)	<0.001	1.44 (1.17, 1.78)	<0.001
*P* for trend	<0.001		<0.001		<0.001	

*NHR, neutrophil to high-density lipoprotein cholesterol ratio; HR, hazard ratio; CI, confidence interval*.

*Model 1: non-adjusted*.

*Model 2: adjusted for age, sex, race, education status, smoker, and alcohol user*.

*Model 3: adjusted for age, sex, race, education status, smoker, alcohol user, diabetes mellitus, hypertension, heart failure, coronary heart disease, stroke, cancer, body mass index, albumin, estimated glomerular filtration rate, antihypertensive drugs, and antidiabetic drugs*.

### Association of NHR With Cardiovascular Mortality

As shown in the Kaplan–Meier survival curves (Figure 2B), there were significant differences in the occurrence of cardiovascular mortality (log-rank test, *p* <0.001) among the tertiles of NHR. After fully adjusting for confounders (Table 2), the *HR* with 95% *CI*s of cardiovascular mortality for participants in the middle and highest tertile compared with those in the lowest participants were 1.30 (1.06–1.59) and 1.44 (1.17–1.78), respectively. The restricted cubic spline curve indicated that NHR was linearly associated with cardiovascular mortality (*p* for non-linearity = 0.553, Figure 3B).

### Subgroup Analysis

As shown in Table 3, a stratified analysis by age, sex, diabetes mellitus, and hypertension, further explored the relationship between NHR and all-cause and cardiovascular mortality. Except for participants <60 years old and individuals with diabetes mellitus, participants in the highest tertile had a higher hazard for all-cause death than those in the lowest tertile (all *p* < 0.05). In addition, there were significant interactions between NHR with sex *(p* for interaction = 0.040) for all-cause mortality that female participants had a stronger association between NHR and all-cause mortality than male participants. Although no significant interactions with NHR for cardiovascular mortality were found, the association between NHR and cardiovascular death was significant among older female people, those with hypertension, and those without diabetes mellitus.

**Table 3:** Subgroup analysis for the association between NHR and cause-specific mortality.

	**Tertile1**	**Tertile2**	**Tertile3**	***P*-t**	***P*-int**
	**HR**	**HR (95%CI)**	**HR (95%CI)**		
All-cause mortality					
Age					0.230
<60 years	Reference	0.96 (0.80−1.16)	1.07 (0.89−−1.29)	0.395	
≥60 years	Reference	1.08 (0.99−1.18)	1.32 (1.20−−1.45)^***^	<0.001	
Sex					0.040
Female	Reference	1.12 (1.00−1.26)^*^	1.45 (1.28−1.64)^***^	<0.001	
Male	Reference	1.01 (0.90−1.13)	1.18 (1.05−1.32)^**^	0.001	
Diabetes mellitus					0.529
No	Reference	1.07 (0.98−1.17)	1.34 (1.22−1.47)^***^	<0.001	
Yes	Reference	1.06 (0.87−1.30)	1.18 (0.97−1.43)	0.070	
Hypertension					0.093
No	Reference	1.01 (0.90−1.14)	1.18 (1.05−1.33)^*^	0.007	
Yes	Reference	1.12 (1.00−1.26)	1.39 (1.24−1.56)^***^	<0.001	
Cardiovascular mortality				
Age					0.147
<60 years	Reference	1.74 (0.94−3.23)	1.49 (0.80−2.80)	0.326	
≥60 years	Reference	1.26 (1.02−1.56)^*^	1.42 (1.14−1.77)^**^	0.002	
Sex					0.768
Female	Reference	1.39 (1.04−1.87)^*^	1.78 (1.29−2.45)^***^	<0.001	
Male	Reference	1.27 (0.95−1.68)	1.31 (0.99−1.74)	0.085	
Diabetes mellitus					0.757
No	Reference	1.34 (1.06−1.68)^*^	1.52 (1.20−1.94)^***^	0.001	
Yes	Reference	1.23 (0.81−1.90)	1.30 (0.86−1.97)	0.250	
Hypertension					0.479
No	Reference	1.25 (0.91−1.71)	1.40 (1.01−1.94)^*^	0.048	
Yes	Reference	1.40 (1.07−1.82)^*^	1.51 (1.16−1.98)^**^	0.004	

*NHR, neutrophil to high-density lipoprotein cholesterol ratio; HR, hazard ratio; CI, confidence interval. Adjusted for age, sex, race, education status, smoker, alcohol user, diabetes mellitus, hypertension, heart failure, coronary heart disease, stroke, cancer, body mass index, albumin, estimated glomerular filtration rate, antihypertensive drugs, and antidiabetic drugs. p-t, p for trend; p-int, p for interaction*.

* *p < 0.05*,

** *p < 0.01*,

*** *p < 0.001*.

## Discussion

To our knowledge, this is the first study to assess the predicative value of NHR for long-term clinical outcomes among the general adult population of the United States. Our results revealed that NHR was independently associated with the risk of mortality from all-cause and CVD. Further analyses suggested that NHR had a non-linear association with all-cause mortality that was more profound among female participants and a linear association with cardiovascular mortality, which remained significant in older female people, those with hypertension, and those without diabetes mellitus.

Recently, increasing numbers of studies have focused on the potential risk markers of long-term clinical outcomes, especially the composite predictors of hematological parameters. These markers derived from routine blood tests are affordable and easily available. Compared with single parameters, the ratio of different parameters can provide comprehensive information and have comparable prediction ability (1). For instance, the ratios of neutrophil to lymphocyte (NLR), platelet to lymphocyte (PLR), monocyte to lymphocyte (MLR), monocyte to HDL-C (MHR), lymphocyte to HDL-C (LHR), triglyceride to HDL-C (THR), and LDL-C to HDL-C are well-studied predictors of mortality and specific-cause mortality (14–20).

Although NHR is a relatively novel indicator, previous studies have found that NHR has a better prognostic value than the above indicators (1, 8, 9). It has several strengths. First, poor outcomes have complicated the pathological processes where both inflammatory reaction and abnormal lipid metabolism are involved. Prognostic indicators, such as NLR, PLR, MLR, THR, and LDL-C/HDL-C could only reflect a single contributor at a time. Nevertheless, NHR can not only simultaneously present the inflammatory status and the lipid metabolism but also indicate the interaction between neutrophils and HDL-C. Second, neutrophils account for the major portion of white blood cells. Accordingly, it had better prediction of cardiovascular mortality and long-term mortality than monocytes and lymphocytes (21–23). Third, neutrophils have been considered as the first line of inflammatory response and are involved in the activation of monocytes and lymphocytes (3, 24). These crucial roles of neutrophils allow NHR to offer a better prognostic potential than MHR or LHR.

However, the prognostic ability of NHR with respect to mortality has not been fully explored. As far as we know, only two previous retrospective studies have explored the prognostic importance of NHR for long-term mortality in specific populations (1, 9). The first study evaluated 528 patients (age range: 65–85 years) with acute myocardial infarction and reported that NHR was a latent predictor of all-cause mortality over a median of 679.50 days, which was superior to MHR and LDL-C/HDL-C (1). The second study followed up 554 patients diagnosed with myocardial infarction with at least one total coronary artery occlusion for over a median of 520 days, and found that higher NHR was associated with increasing risk of cardiovascular mortality. In addition, NHR offered better prediction than other ratios, such as PLR, NLR, MHR, THR, LHR, and HDL-C/LDL-C (9). In accordance with these studies, we demonstrated that NHR was a potential prognostic marker of all-cause mortality and cardiovascular mortality in the general population over a longer follow-up.

Several interpretations can account for the correlation between higher NHR and poorer prognosis. Neutrophils were abundantly identified in the advanced atherosclerotic plaques and its count was positively associated with the histopathologic features of rupture-prone atherosclerotic lesions (25). The preformed granule proteins released from neutrophils, such as myeloperoxidase (MPO) and matrix metalloproteinases, contribute to severe inflammatory function that precede myocardial injury (25, 26). Moreover, the neutrophil activation and consecutive interactions with platelets contribute to atherothrombosis, thereby causing cardiovascular mortality (27). Activated neutrophils can also mediate HDL oxidation and impair cholesterol efflux capacity by possessing oxidant-generating enzymes, such as MPO, NADPH oxidase, and nitric oxide synthase (28). By contrast, HDL-C inhibits neutrophil activation, adhesion, proliferation, and migration, and anti-inflammatory effect was strongly associated with the abundance of lipid rafts (29). In addition, HDL-C can promote the angiogenesis on endothelial cells and inhibit apoptosis on cardiomyocytes to exert cardioprotective effects (30). Previous studies have shown that the higher NHR was associated with severe stroke characterized by higher mortality (8) and more culprit lesions that could cause more myocardial damage and increased cardiovascular mortality (9). Above all, this is explainable that the increased NHR attributed to both the increased neutrophil count and decreased HDL-C level was associated with poorer outcomes.

Our study added a novel insight in that there was a non-linear association between NHR and all-cause mortality, although the underlying mechanism remains unclear. One plausible explanation is that the concentrations of HDL-C were higher among participants with lower NHR in this cohort. In fact, recent epidemiological studies found that HDL-C was related to all-cause mortality in a U-shaped pattern such that both the lower and higher HDL-C concentrations could increase the risk of all-cause mortality (31–33). Huang et al. found that HDL-C had a concentration-related biphasic effect and could lost protective effect at high concentrations (34). Moreover, genetic variants may also play an adverse role in the health status and lead to increased risk of mortality among individuals with high level of HDL-C (35). In addition, we noted that the average age and the prevalence of cancer were higher among those with lower NHR, suggesting perhaps that age and cancer are likely to explain the increased risk of death in this group. Thus, additional investigation is required to confirm the influence of age and cancer on the association between lower NHR and increased risk of all-cause mortality.

Our study has several strengths. First, we provided more evidence than is currently available about the prognostic role of NHR for all-cause and cardiovascular mortality in the general population from a large-scale, prospective cohort study. Second, we performed both qualitative and quantitative analyses to explore the relationship between NHR and survival. Last, we showed the shape of the relationship of NHR with long-term outcomes *via* the restricted cubic spline model for the first time.

Our study has some limitations. First, NHR was only measured at baseline, which might not reflect the time-dependent association of NHR with long-term outcomes. Second, some variables, such as lifestyle factors and medical history were self-reported, which might have caused recall bias. Third, although these results were adjusted for covariates as far as possible, we could not exclude the possible residual confounding effects of unmeasured or non-included variables. Fourth, there were no direct comparisons with other inflammatory indexes, such as C-reactive protein, because of the limited data of the NHANES. Last, our data were derived from the U.S. database, so it may not be generalizable to other regions or populations. Thus, more epidemiologic studies are needed to establish the predictive value of NHR in clinical practice.

## Conclusions

In summary, our study indicated that NHR was independently associated with all-cause and cardiovascular mortality in the general population, which supported that NHR might be a potential predictor of long-term clinical outcomes.

## Data Availability Statement

The original contributions presented in the study are included in the article, further inquiries can be directed to the corresponding author.

## Ethics Statement

The studies involving human participants were reviewed and approved by the Ethics Review Board of National Center for Health Statistics. The patients/participants provided their written informed consent to participate in this study.

## Author Contributions

MJ contributed to conceptualization, formal analysis, and writing-original draft of the manuscript. JS responsible for software and data curation. HZ performed visualization. ML and ZS contributed to validation. WS performed writing–review and editing. XK contributed to conceptualization and supervision. All authors contributed to manuscript revision and reading and approved the submitted version.

## Conflict of Interest

The authors declare that the research was conducted in the absence of any commercial or financial relationships that could be construed as a potential conflict of interest.

## Publisher's Note

All claims expressed in this article are solely those of the authors and do not necessarily represent those of their affiliated organizations, or those of the publisher, the editors and the reviewers. Any product that may be evaluated in this article, or claim that may be made by its manufacturer, is not guaranteed or endorsed by the publisher.
